# Genome Sequence of Kurthia Type Species Kurthia zopfii Strain ATCC 33403^T^

**DOI:** 10.1128/MRA.00833-18

**Published:** 2018-07-12

**Authors:** Abigail E. Goen, Kyle S. MacLea

**Affiliations:** a Biology Program, University of New Hampshire, Manchester, New Hampshire, USA; b Biotechnology Program, University of New Hampshire, Manchester, New Hampshire, USA; c Department of Life Sciences, University of New Hampshire, Manchester, New Hampshire, USA; University of Delaware

## Abstract

The genome of the type strain of the Kurthia genus, Kurthia zopfii ATCC 33403, was sequenced. Nonpathogenic K. zopfii has been isolated from intestinal contents, fecal material, meats, meat products, milk, water, and air, including air at high altitudes. The predicted genome size is 2,878,279 bp, with 37.05% G+C content.

## Abstract (precis)

The genome of the type strain of the Kurthia genus, Kurthia zopfii ATCC 33403, was sequenced. Nonpathogenic K. zopfii has been isolated from intestinal contents, fecal material, meats, meat products, milk, water, and air, including air at high altitudes.

## ANNOUNCEMENT

Characteristically, the phylum *Firmicutes* consists of Gram-positive bacteria with low G+C content. In class Bacilli and order Bacillales, the family Planococcaceae includes the genus Kurthia, of which Kurthia zopfii is the type species and strain ATCC 33403 (= BARNES F64/100; DSM 20580; NCIB 9878; and NCTC 10597) is the type strain, which was first isolated in 1883. K. zopfii is a motile, saprophytic, Gram-positive firmicute of regular, unbranched rods that fragment into a coccoid morphology in older cultures (1–4). The type strain of K. zopfii was isolated from intestinal contents of poultry (5) as “Bacterium zopfii,” and many strains have since been found in meats, meat products, and milk, air from abattoirs, and also environments as diverse as wastewater and air samples collected above 3,000 m in altitude (1–4, 6–10). Although some members of the genus Kurthia are pathogens (11–13), no reports of pathogenicity have been made for K. zopfii.

The type strain of K. zopfii, ATCC 33403 (Kurth 1883) Trevisan 1885, was ordered (ATCC, Manassas, VA, USA) and rehydrated. Reconstituted bacteria were spread on tryptic soy agar (TSA) incubated at 26°C and then streaked onto brain heart infusion (BHI) agar for isolates. A single colony was then used to inoculate BHI broth, which was used to generate genomic DNA (gDNA) with a DNA minikit (Qiagen, Valencia, CA, USA). The double-stranded DNA (dsDNA) broad-range assay kit (DeNovix, Wilmington, DE, USA) was used to check DNA quality and quantity. One nanogram of clean gDNA was fragmented and tagged with adapters with the HyperPlus kit (KAPA, Wilmington, MA, USA), and ∼5% of the total lane yield of 11 pM of a gDNA library was loaded on an Illumina HiSeq 2500 instrument by the Hubbard Center for Genome Studies (Durham, NH, USA) for sequencing. Prior to genome assembly, 250-bp paired-end reads were trimmed using Trimmomatic (14). We used SPAdes v. 3.11.1 (15) to assemble the genome using default settings. After removal of small contigs, the genome was submitted for annotation via the NCBI Prokaryotic Genome Annotation Pipeline (PGAP) process (16). The K. zopfii genome-sequencing project produced 110 contigs with a total length of 2,878,279 bp. The largest contig was 204,291 bp, with an *N*_50_ value of 59,099 bp and a G+C content of 37.05%. PGAP predicted 2,925 genes, of which 2,825 were protein coding genes, along with 13 rRNAs, 53 tRNAs, 5 other RNA genes, and 29 pseudogenes. The genome size is smaller than that of Kurthia sibirica, at 3.5 Mbp and 3,400 total genes (17). Although we achieved 344× genome coverage, it is possible that the K. zopfii draft genome is incomplete, resulting in an undercount of genes versus that of other members of the genus. Alternatively, these species may be quite different given their reportedly low DNA-DNA hybridization results (∼20%) (18). Average nucleotide identity (ANI) scoring using the EzBioCloud portal (19) generated 74.5% ANI, consistent with assignment to the same genus, although K. zopfii and K. sibirica are clearly quite different members of the *Kurthia* genus.

### Data availability.

The Kurthia zopfii ATCC 33403^T^ whole-genome shotgun sequence (WGS) project has been deposited in DDBJ/ENA/GenBank under accession number QFVS00000000. The version described in this paper is the first version, QFVS01000000.
